# Engineering-Oriented Genetic Algorithm Framework for Ultra-Broadband Metasurface Absorber Inverse Design

**DOI:** 10.3390/ma19183930

**Published:** 2026-09-16

**Authors:** Lejia Wu, Dawei Zhang

**Affiliations:** Engineering Research Center of Optical Instrument and Systems, Ministry of Education and Shanghai Key Lab of Modern Optical System, University of Shanghai for Science and Technology, Shanghai 200093, China

**Keywords:** improved genetic algorithm, metasurface absorber, ultra-broadband, FDTD, nanostructure array

## Abstract

This paper proposes an engineering-oriented inverse-design framework to engineer metasurface nanostructures for ultra-broadband electromagnetic absorption over 0.36–10.36 μm. The framework integrates a genetic algorithm (GA) with finite difference time domain (FDTD) simulation and uses a composite objective function combining full-spectrum average reflectance and absorptivity standard deviation to balance absorption level and spectral uniformity. Three strategies, namely dynamic elitist preservation, fitness reuse, and diversified candidate expansion, are introduced to improve the search ability, reduce redundant FDTD evaluations, and retain multiple engineering-relevant candidate solutions. The optimized structure exhibits an average absorptivity of 90.9%, a standard deviation of 0.054, a coefficient of variation of 0.059, a deviation of 0.044, and a Relative Deviation of 0.049. The absorber also maintains robust performance under different polarization states and oblique incidence; over 0–55°, the average absorptivity is 88.9% for TE polarization and 92.5% for TM polarization, with an overall unpolarized average absorptivity of 90.7%. These results demonstrate a design workflow that extends GA inverse design from searching for a single minimum-fitness structure toward engineering-oriented multi-criteria candidate selection.

## 1. Introduction

Metasurfaces are two-dimensional arrays of subwavelength elements that enable flexible manipulation of electromagnetic waves through the structural design of their constituent meta-atoms. Their diverse functionalities have led to broad applications in polarization control, beam shaping, imaging, sensing, and light–matter interaction. Plasmonic materials are particularly important in metasurface research because of their strong interaction with electromagnetic fields at subwavelength scales. Recent studies have investigated plasmonic structures based on one-dimensional photonic crystals, particle-based and structurally varying systems, and multilayer thin-film architectures, providing diverse approaches for controlling and manipulating light [1,2,3,4].

Metasurface absorbers (MAs) are typical subwavelength electromagnetic (EM) devices. They have attracted extensive attention due to their ultra-thin configurations and strong EM wave absorption. These devices achieve interface impedance matching via artificially designed subwavelength resonant units, which suppress reflection and enhance EM dissipation. MAs have been widely applied in stealth technology, filtering, infrared detection, and solar-energy harvesting [5,6,7,8,9,10]. From the perspective of surface engineering, metasurface absorbers represent a promising candidate for next-generation thin-film electromagnetic absorption coatings, with practical application scenarios covering aircraft infrared suppression, electronic-device electromagnetic interference shielding, and thermal radiation management of high-temperature components. Broadband performance is increasingly critical with the rapid expansion of application scenarios. Broadband MAs extend the working spectrum and improve frequency drift tolerance. They offer irreplaceable merits for multi-band stealth and broadband energy harvesting. Accordingly, ultra-thin broadband MAs have emerged as a research hotspot in electromagnetics and photonics [11,12,13,14,15,16,17].

Methods for bandwidth broadening include multi-resonance coupling, multilayer stacking, and hybrid structures. These approaches extend the absorption bandwidth, but most rely on empirical trial and error and massive parameter sweeps. The nonlinearity of parameter coupling within complex structures results in low search efficiency for traditional design strategies. Conventional methods can hardly fully explore optimal solutions in the parameter space, so there remains room for improvement in bandwidth and absorptivity. Intelligent optimization algorithms offer an effective way for the inverse design of MAs [18,19,20,21,22,23].

The genetic algorithm requires no gradient information and can efficiently explore high-dimensional parameter spaces. Combined with full-wave simulation, it can obtain non-intuitive optimal structures that are hard to achieve through manual design [2,24]. However, current GA-based metasurface designs still suffer from several limitations. The algorithm is often treated merely as a parameter-tuning tool without targeted refinements or analysis of how objective functions guide the optimization process and final outcomes. Performance evaluation usually adopts limited evaluation metrics, leading to an incomplete assessment of device operational performance, especially when evaluating the practical suitability for surface-coating-oriented usage that requires angular robustness and spectral stability.

This work presents an optimized design approach that integrates an improved GA with electromagnetic simulation to address the aforementioned limitations, aiming to establish an algorithm-driven design workflow for ultra-broadband metasurface absorbing coatings. This method achieves global parametric optimization across the 0.36–10.36 μm band. A composite objective function is constructed using full-spectrum average reflectance and absorptivity standard deviation. Its guiding effect on balanced broadband absorption is analyzed, and a multidimensional quantitative evaluation system is established to comprehensively characterize overall absorption performance. Three enhancement strategies, including elitist preservation, fitness reuse, and diversified candidate expansion, are introduced to strengthen the search ability, stability, and efficiency of the algorithm. The polarization insensitivity and angular stability of the device are quantitatively assessed under diverse polarization states, varying incident angles, and different azimuthal orientations, which are key prerequisites for real-world coating applications on non-planar substrates. The physical mechanism of broadband absorption is explained from the perspectives of impedance matching, providing theoretical foundations for the design of metasurface-based absorbing coating surfaces.

## 2. Materials and Methods

**Figure 1:**
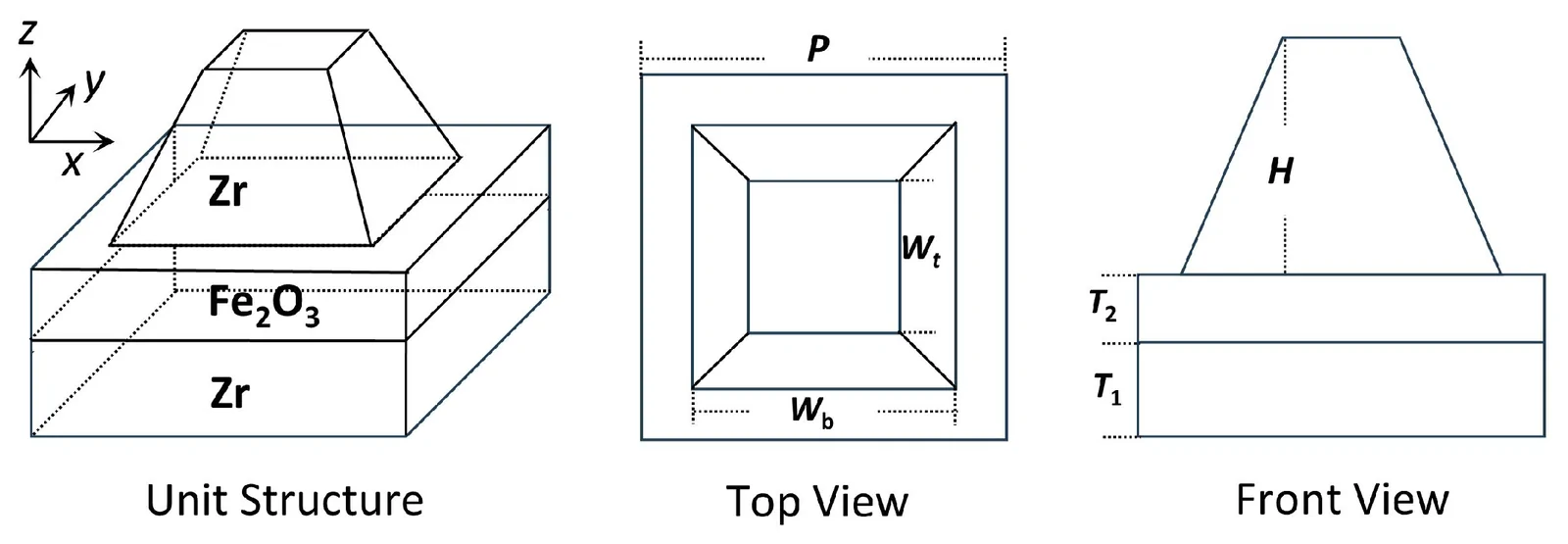
Schematic diagram of the MA unit structure.

The ultra-broadband MA is constructed based on a periodic unit array. Each unit adopts a three-layer stacked configuration consisting of a top metallic pyramid pattern layer, a middle dielectric layer, and a bottom metallic substrate. The schematic structure is shown in Figure 1. The bottom width, top width, and vertical height of the pyramid are defined as *W_b_*, *W_t_*, and *H*, respectively. The thicknesses of the substrate layer and middle dielectric layer are denoted as *T*_1_ and *T*_2_, respectively. The dielectric layer is made of iron sesquioxide (Fe_2_O_3_), while the pattern layer and the substrate consist of metallic zirconium (Zr). The optical constants of Zr and Fe_2_O_3_ used in this study are taken from reported experimental data, and the adopted data cover the complete investigated wavelength range of 0.36–10.36 μm [25]. No additional fitting is introduced to fill a missing part within the investigated band.

In this work, the FDTD method is adopted to calculate the EM responses of the structure and integrated with an improved GA to implement global optimization for the unit geometric parameters. Three-dimensional structure modeling and EM simulations are conducted on the Lumerical 2026 R1.3 platform. All simulations use a unified basic configuration. Non-optimized parameters are fixed to exclude interference of irrelevant variables on optimization results. *T*_1_ is set to 200 nm, while the unit period (*P*), lateral widths of both the substrate and middle layers, and simulation domain size are fixed at 360 nm. The excitation source is a normally incident plane wave propagating along the negative z-axis. The wavelength range covers 0.36–10.36 μm. An automatic non-uniform mesh with an accuracy of 2 is adopted for the simulation. Periodic boundary conditions are applied in the *x* and *y* directions, while perfectly matched layers (PMLs) are set at the upper and lower boundaries along the z direction.

**Figure 2:**
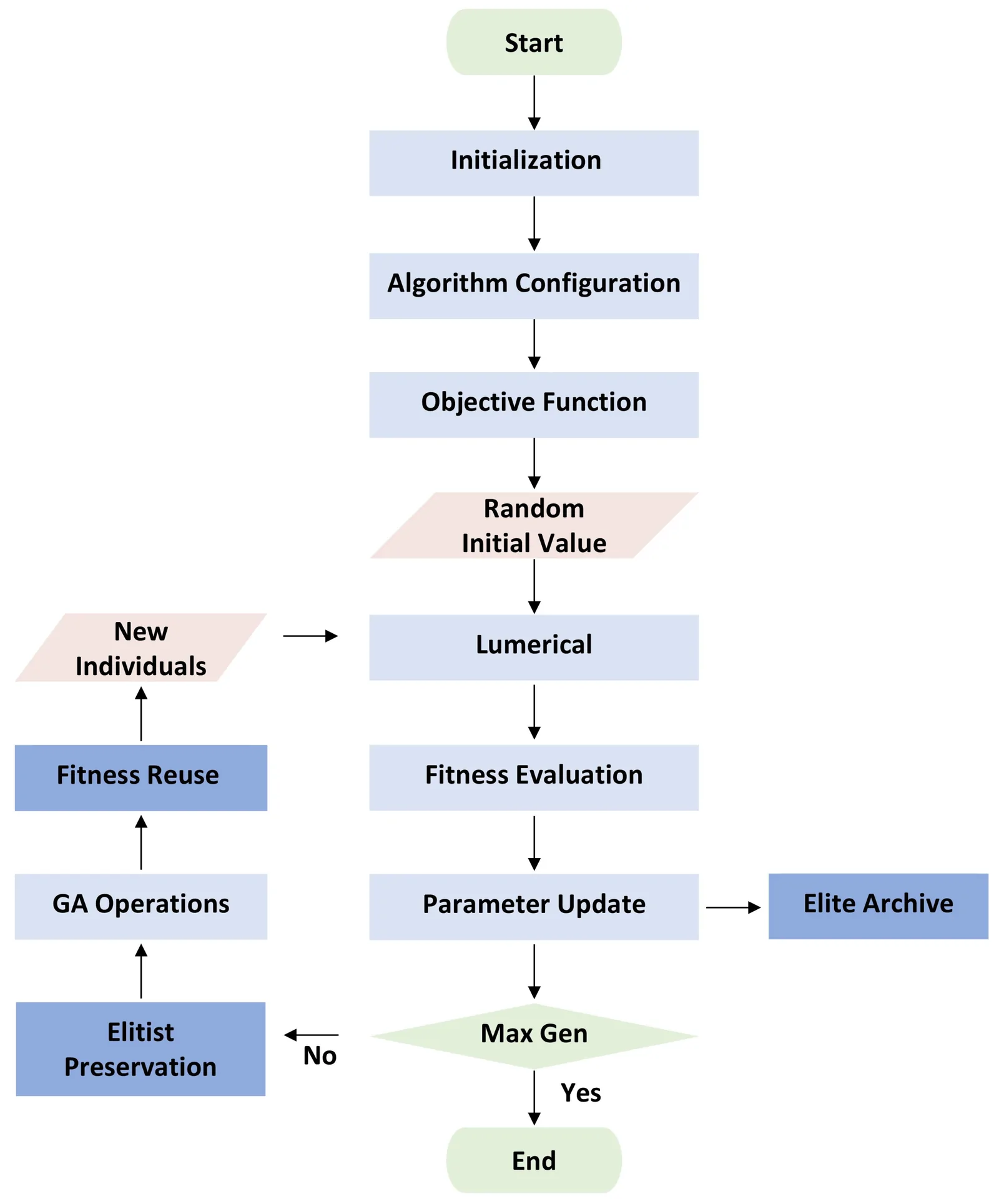
Flowchart of the improved GA optimization.

The optimization is carried out via the joint operation of Python 3.14.7 and Lumerical 2026 R1.3. The complete workflow is shown in Figure 2. The overall process proceeds as follows: after initializing the population and defining the algorithm hyperparameters and objective functions, the program generates a set of individuals with random geometric parameters. These parameters are imported into Lumerical for FDTD simulation. Subsequently, the fitness values are calculated and stored. Next, the program updates the parameters and saves superior individuals of each generation into an elite archive. Eventually, the program checks whether the iteration count reaches the preset maximum generation. If the termination condition is not met, the algorithm performs elitist preservation, genetic operations, and fitness reuse in sequence. New individuals to be evaluated are generated, and simulation and evaluation are conducted iteratively. The optimization process terminates when the iteration reaches the preset maximum generation.

More specifically, the GA code is executed in PyCharm 2026.2.2 using Python, and Lumerical FDTD is called through its Python API interface. Python handles population initialization, objective-function evaluation, selection, crossover, mutation, elitist preservation, fitness reuse, and candidate screening, while Lumerical performs the three-dimensional structure construction and full-wave electromagnetic simulation. Geometric parameters and simulation results are transferred between the two environments during the optimization loop. To make the fitness-reuse criterion unambiguous, the optimized geometric parameters are stored with a fixed numerical precision of two decimal places; the identical parameter vectors represent identical simulated geometries.

The absorptivity of the MA is given by Equation (1) [26,27]. Because the 200 nm Zr substrate suppresses transmission over the investigated spectral range, *T*(*λ*) can be approximated as zero. Accordingly, *A*(*λ*) ≈ 1 − *R*(*λ*) [27] for the present structure, and minimizing reflectivity provides the main route to high absorption.

(1) *A*(*λ*) = 1 − *R*(*λ*) − *T*(*λ*)

where *A*(*λ*), *R*(*λ*), and *T*(*λ*) represent the absorptivity, reflectivity, and transmissivity at wavelength *λ*, respectively.

The geometric variables to be optimized are *W_b_*, *W_t_*, *H*, and *T*_2_. The key hyperparameters are set as follows: crossover probability = 0.5, mutation probability = 0.2, population size = 50, and maximum generations = 30. This optimization is a multi-parameter and multi-objective problem. Each wavelength corresponds to an independent absorption constraint. Directly taking each wavelength point as an optimization target will substantially raise the optimization complexity owing to the ultra-broad operating bandwidth. Accordingly, full-spectrum average reflectivity, $\bar{R}$, and absorptivity standard deviation, *σ_A_*, are introduced to construct the objective function, as expressed in Equation (2). The algorithm aims to minimize the objective function.

(2) $$f=\beta \cdot \bar{R}+\gamma \cdot \sigma_{A}$$

Three groups of comparative weights are set as follows:

(a) *f*_1_: *β* = 1, *γ* = 0;
(b) *f*_2_: *β* = 0, *γ* = 1;
(c) *f*_3_: *β* = 1, *γ* = 1.

Three improved strategies are introduced to strengthen algorithm-search performance and optimization stability, which can elevate operational efficiency, cut computational costs, expand candidate solutions, and ensure reliable optimization results.

(1) Dynamic Elitist Preservation Strategy

The GA completes population iteration through selection, crossover, and mutation operations. Random operations may cause the loss of optimal individuals. The elitist preservation strategy preserves superior individuals and directly passes them to the next generation, thereby avoiding population degradation. A segmented dynamic elitist preservation rule is adopted. Five optimal individuals are retained per generation in the first 20 generations, and ten optimal individuals per generation in the last 10 generations. A small number of elites in the early stage maintains population diversity and fully explores the parameter space, helping prevent algorithm premature convergence. More elites in the later stage strengthen local fine search and ensure stable convergence to optimal solutions. The segmented mechanism balances the algorithm’s global exploration and local exploitation, thereby enhancing its optimization efficiency and convergence stability.

(2) Fitness Reuse Strategy

Selection, crossover, and mutation constitute the core evolutionary operators of GA. These operators frequently generate duplicate individuals throughout iterations. To address this issue, a fitness reuse mechanism is introduced into the GA. If a new individual shares identical parameters with any previously simulated individual, its fitness value is directly retrieved from the archive, avoiding redundant FDTD calculations. This strategy drastically cuts repetitive simulations, reducing total computational cost and accelerating optimization.

(3) Diversified Candidate Expansion Strategy

Driven by the objective function, the GA navigates the parameter space to seek minimal fitness solutions. Most objective functions are constructed based on a single evaluation indicator, such that the solution with optimal fitness cannot guarantee satisfactory holistic performance. Given that classic GAs preserve one elite individual with the lowest fitness, promising candidates with practical engineering practicability are inevitably overlooked. The improved GA contains a diversified candidate expansion mechanism to make up for the limitation of single solution output. Superior individuals of each generation are stored in the elite archive in the order of fitness. After the iteration, differentiation screening is then performed based on the L1 distance threshold. The process finally retains 10 candidate parameter combinations with high distinctiveness. FDTD simulations are conducted for all candidate configurations to select the structure with the best holistic performance.

Elite individual diversity screening relies on the threshold method based on L1 distance. The threshold-based screening rule in this work is defined in Equations (3)–(5).

(3) $$\left\{\begin{array}{c} \tau_{i}=\delta \cdot \left({ub}_{i}-{lb}_{i}\right)\\ \tau_{total}=\sum_{i=1}^{4}\tau_{i} \end{array}\right.$$

The single-dimensional threshold, *τ_i_*, is calculated based on the bounds of each parameter with a preset ratio, *δ* = 0.05. The total threshold, *τ*_total_, is accumulated by summing all single-dimensional thresholds. A relaxation coefficient, *ζ*, with values of 0.2, 0.1, and 0 is adopted to dynamically adjust the screening threshold. The dynamic threshold is denoted as *τ*_dyn_.

(4) $$\tau_{dyn}=\tau_{total}\cdot \zeta$$

The L1 distance between two parameter vectors, ***m*** and ***n***, is defined by Equation (5).

(5) $$d_{L_{1}}\left(m,n\right)=\sum_{i=1}^{4}\left|m_{i}-n_{i}\right|$$

An individual is recognized as a valid and distinctive candidate and added to the candidate set if its minimum L1 distance to the selected set is larger than the current threshold. The algorithm relaxes the threshold step by step. It steadily outputs 10 optimal and distinctive geometric parameter combinations.

## 3. Results

### 3.1. Optimization Results and Performance

Multiple comparative simulation experiments are conducted in this section based on the optimization framework in Section 2. The optimization performance of the improved GA is systematically evaluated from the perspective of objective function. Both the algorithm effectiveness and the comprehensive properties of the MA are quantitatively assessed via a multidimensional evaluation system.

**Figure 3:**
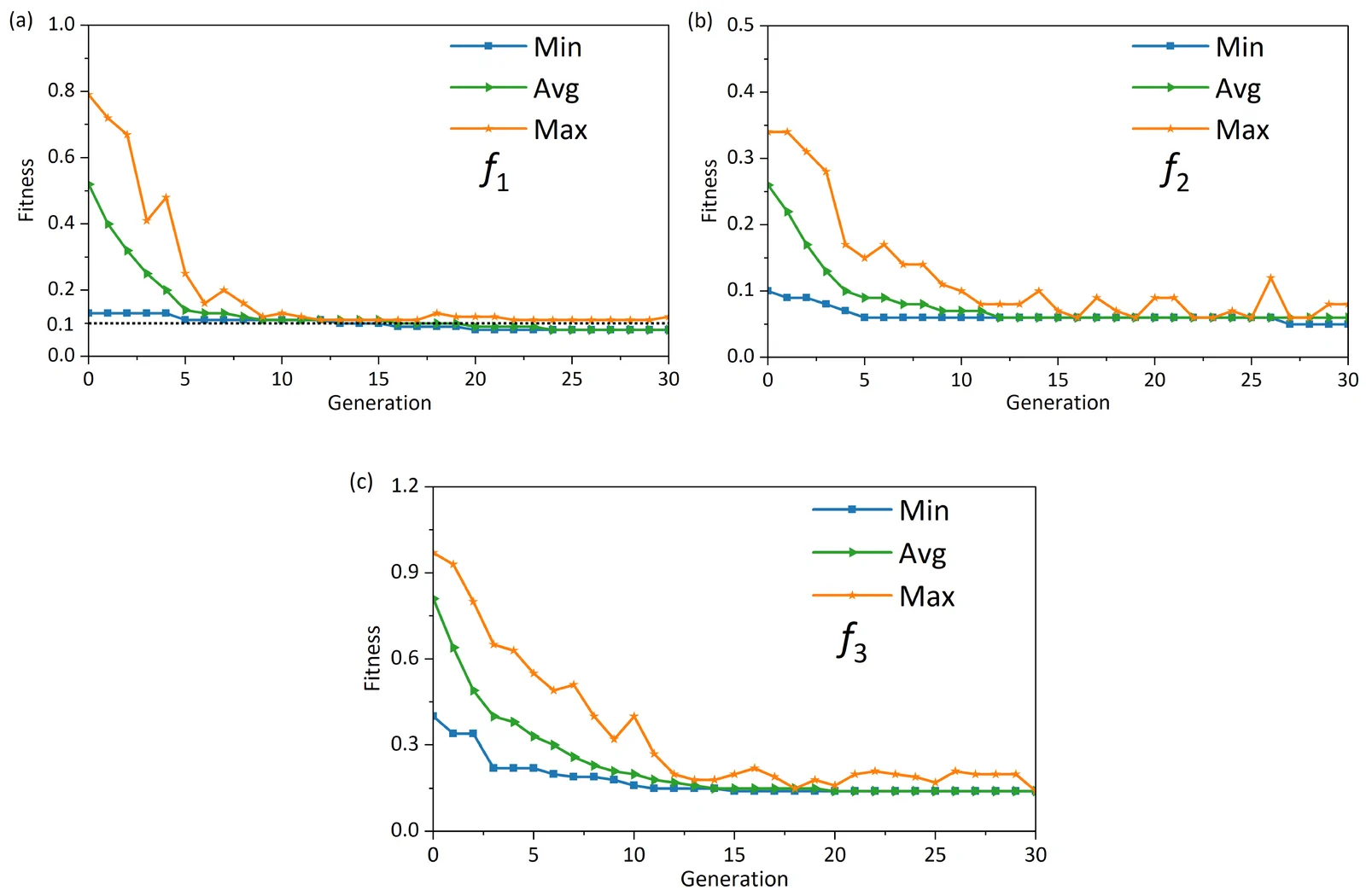
Convergence curves of the improved GA under different objective functions. Min, Avg, and Max represent the minimum, average, and maximum fitness values of each generation, respectively. (**a**) *f*_1_, (**b**) *f*_2_, and (**c**) *f*_3_.

The objective function provides core guidance for iterative optimization in the GA. The optimization performances of three objective functions, namely *f*_1_, *f*_2_, and *f*_3_, are compared, including analyses of algorithm convergence, candidate geometric parameter distributions, and absorption spectral characteristics. Figure 3 shows convergence curves of the improved GA under different objective functions. Results show that the algorithm converges steadily under all objective functions. The final minimum fitness values are 0.08 for *f*_1_, 0.05 for *f*_2_, and 0.14 for *f*_3_.

**Figure 4:**
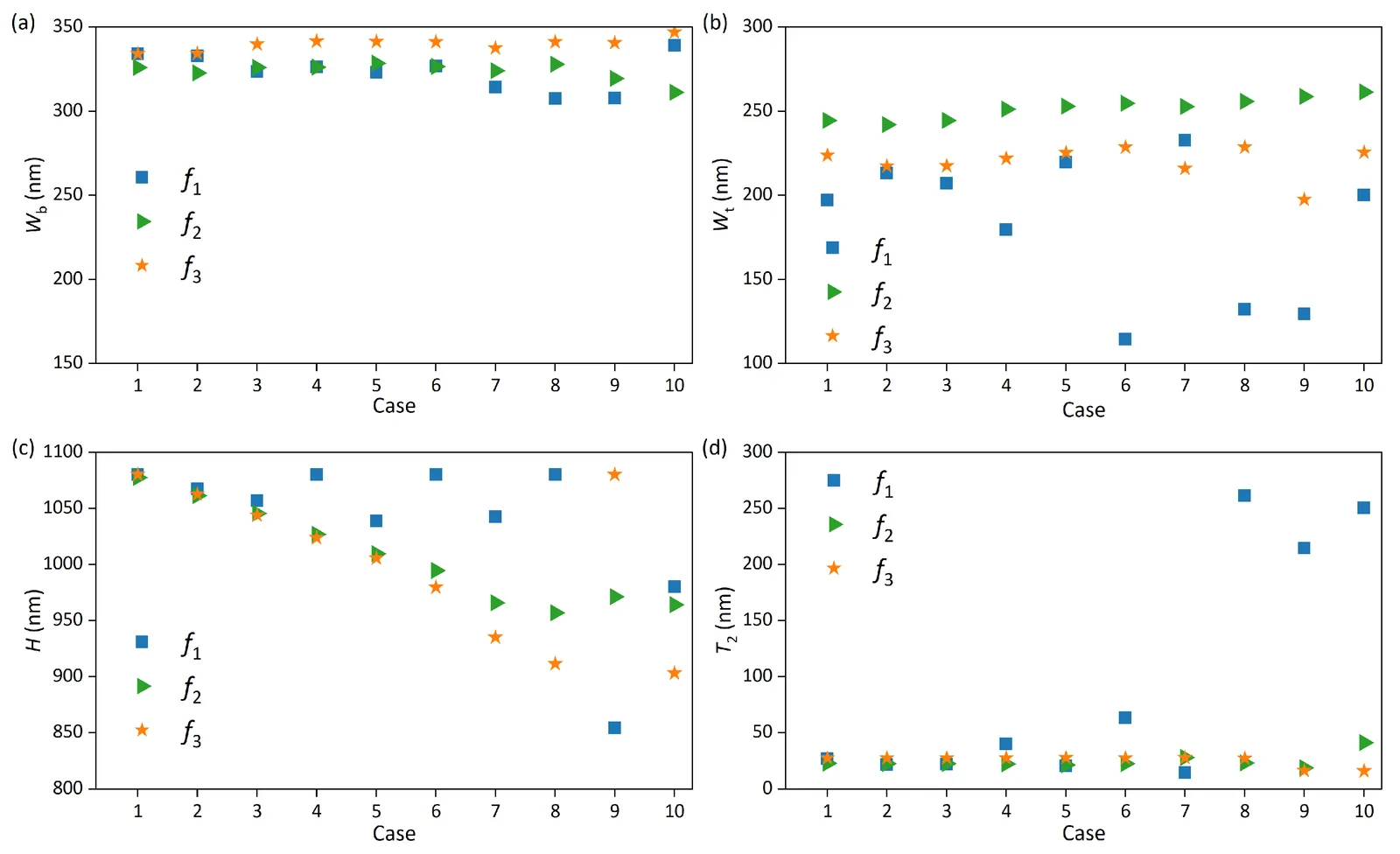
Geometric parameter distributions of the candidate individuals optimized by different objective functions. (**a**) *W_b_*, (**b**) *W_t_*, (**c**) *H*, and (**d**) *T*_2_.

After the diversified screening operation, each objective function yields 10 distinct candidate individuals. These individuals are labeled Case 1 to Case 10 in ascending order of fitness. Their corresponding geometric parameter distributions are shown in Figure 4.

Parameter distributions show that the bottom width, *W_b_*, of all individuals concentrates in a narrow range of 300–350 nm, with small differences. The top width, *W_t_*, distribution is significantly affected by the objective function. It shows an overall increasing trend as the weight of the standard deviation term rises. The underlying mechanism is that the standard deviation term suppresses spectral fluctuations and flattens the absorption spectrum, while a proper increase in *W_t_* will improve spectral uniformity. The pyramid height, *H*, exhibits a scattered distribution, and its value gradually converges as the fitness value declines. The parameters of optimal individuals under different objective functions tend to be consistent. This pattern indicates that the effects of *H* on absorption performance are more linear. The dielectric thickness, *T*_2_, of most individuals is distributed within 0–50 nm, which implies that its influence on absorption remains relatively stable.

**Figure 5:**
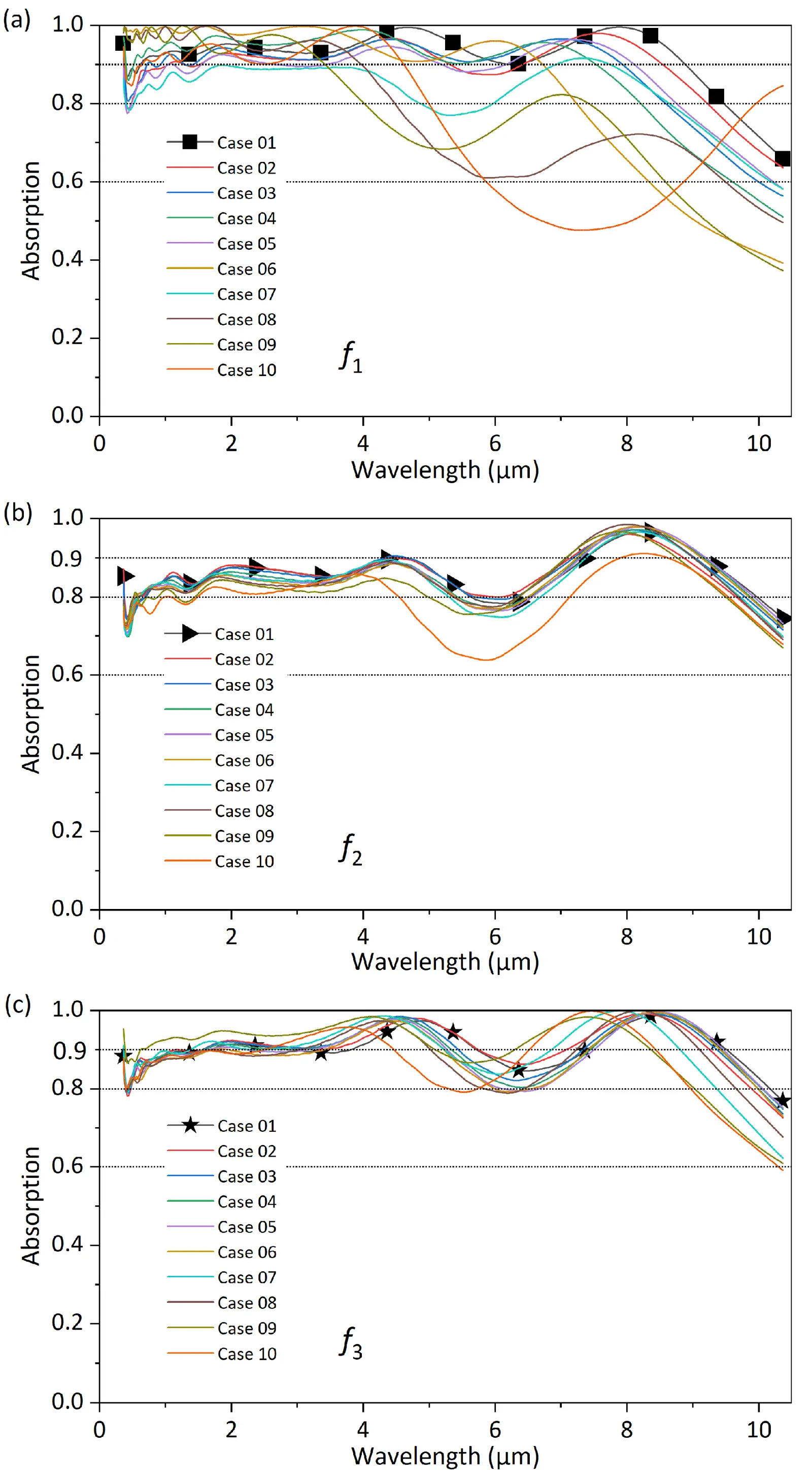
Broadband absorption spectra of the candidate individuals optimized by different objective functions. (**a**) *f*_1_, (**b**) *f*_2_, and (**c**) *f*_3_.

FDTD simulations provide ultra-broadband absorption spectra of the candidate individuals. Results are presented in Figure 5. The spectral characteristics show several clear trends. Optimization based only on minimizing average reflectivity (*f*_1_) yields high average absorptivity yet poor spectral uniformity. Short-wave absorption is relatively high, while long-wave absorption drops significantly. Optimization based only on standard deviation (*f*_2_) greatly improves spectral flatness but fails to guarantee high absorption. The absorptivity at most frequency points ranges from 0.8 to 0.9, and the absorptivity within the main operating band of 0.36–7 μm stays below 90%.

The hybrid objective function yields absorption spectra featuring both high absorptivity and good flatness. The drop in long-wave absorptivity is significantly suppressed compared to the single average objective. The overall absorption level is greatly improved compared with the single standard deviation objective. The proportion of wavelengths with absorptivity above 90% increases notably in the band of 0.36–7 μm. The algorithm achieves balanced trade-off among broadband response, high absorptivity, and low absorption fluctuation. These comparative results confirm that the objective function determines the optimization direction and dominates the final device performance. The objective function ought to be flexibly chosen according to practical engineering design demands.

A single fitness value cannot fully characterize the comprehensive performance of an ultra-broadband MA. In this study, a multidimensional evaluation system is established for quantitative comparison and standardized assessment of different optimization schemes. The system contains the following metrics:

(a) Full-spectrum average absorptivity ($\bar{A}$).
(b) Standard deviation of absorptivity ($\sigma_{A}$) and coefficient of variation (CV), which characterize absorption fluctuation.
  (6) $$CV=\sigma_{A}/\bar{A}$$
(c) High-absorption ratio (HAR), defined as the proportion of sampling points with absorptivity ≥ 90% across the 0.36–10.36 μm band.
(d) Quantification metrics for evaluating the deviation level of spectral bands with absorptivity lower than 90%. These metrics cover Absolute Deviation (AD), Mean Absolute Deviation (MAD), Root Mean Square Error (RMSE), Relative Deviation (RD), and Mean Relative Deviation (MRD). They quantitatively describe spectral deviations from distinct perspectives.

(7) $$AD=\sum \left|A_{i}-A_{ref}\right|$$

(8) $$RD=\left|A_{i}-A_{ref}\right|/A_{ref}$$

where $A_{i}$ represents the absorptivity of the deviation point, and *A*_ref_ = 0.9.

The absorption evaluation quantities, HAR, AD, MAD, RMSE, MRD, and RD, used in this study are defined as engineering metrics in this work. Standardized comparisons and performance evaluations of optimization results under different objective functions are carried out based on the quantitative system. Multidimensional quantitative results in Table 1 exhibit distinct performance disparities. The single average objective achieves the best $\bar{A}$ and HAR, yet strong absorption attenuation in the long-wave band induces large fluctuations and inferior uniformity relative to the standard deviation objective. The single standard deviation objective significantly flattens the absorption curve but fails to improve overall absorption, with an HAR of merely 0.204.

The hybrid objective achieves collaborative optimization of high absorption and spectral uniformity. It obtains a better balance among multiple indicators. The average absorptivity of the hybrid scheme reaches 90.6%. This value is slightly lower than the 92.1% achieved by the average scheme but higher than the 86.2% of the standard deviation scheme. The HAR of the hybrid scheme is lower than that of the average scheme but greatly improved compared with the standard deviation scheme. The hybrid scheme also exhibits superior spectral uniformity. Its fluctuation metrics are lower than those of both single-objective schemes. The $\sigma_{A}$ and CV reach only 0.046 and 0.051, respectively. Furthermore, the hybrid objective function achieves the optimal performance in absorption deviation assessment, with all deviation metrics outperforming those of single-objective schemes. The AD, MAD, RMSE, MRD, and RD_max_ are 7.483, 0.032, 0.043, 0.035, and 0.146, respectively. These low values indicate minor deviation magnitudes, verifying that the overall performance satisfies the demands of practical engineering applications.

In summary, optimization guided by average reflectivity improves overall absorption but cannot ensure flat spectra. Optimization guided by absorptivity standard deviation improves uniformity but does not significantly raise the absorption. The hybrid objective function balances absorptivity, bandwidth, spectral fluctuation, and absorption deviation. It enables coordinated optimization of multiple performance indicators and provides a practical design scheme for ultra-broadband MAs.

### 3.2. Validation of Enhancement Strategies

**Figure 6:**
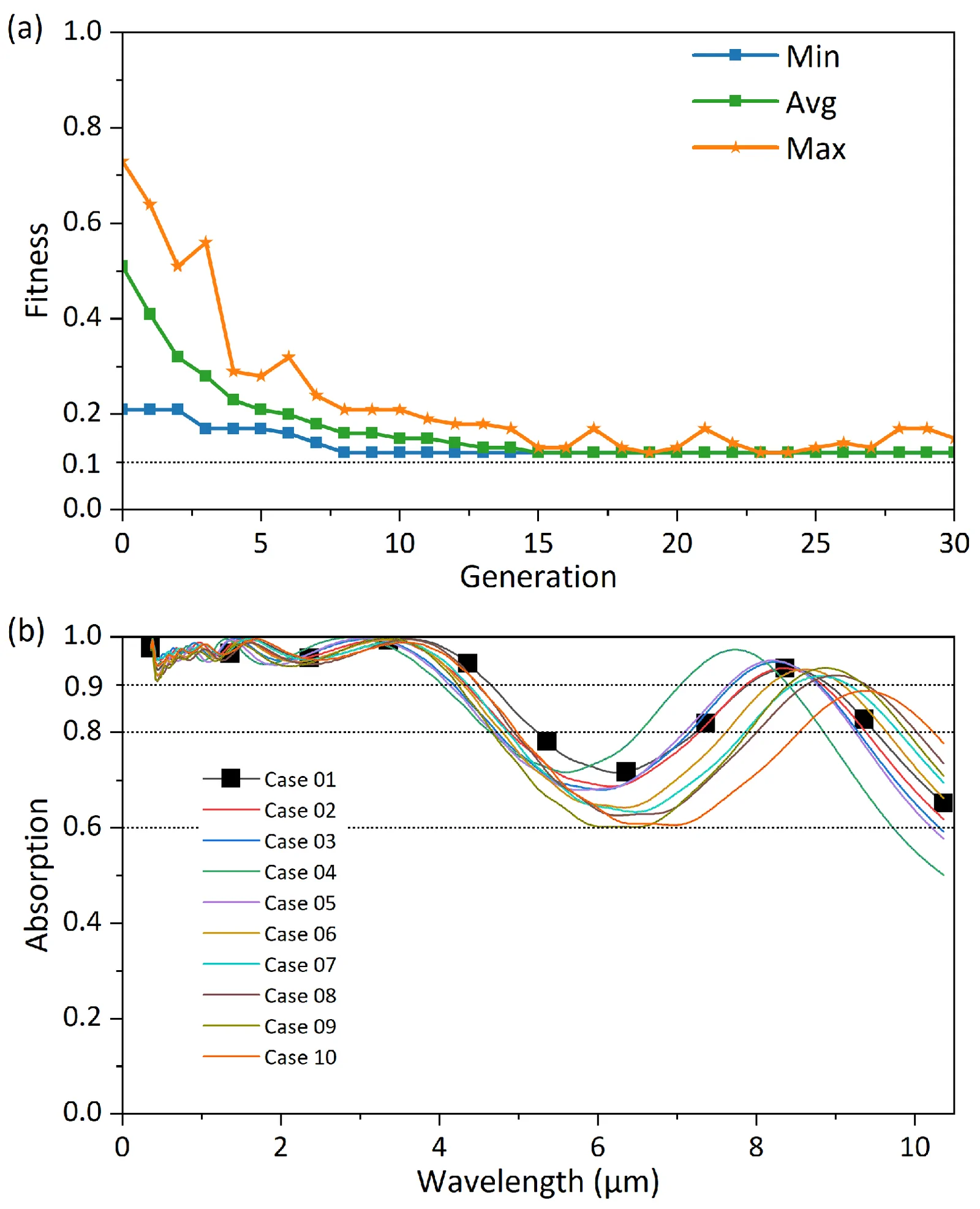
Optimization results of objective function *f*_1_ without the elitist preservation strategy. (**a**) Convergence curve. (**b**) Broadband absorption spectra of the candidate individuals.

The improved GA integrates three strategies, namely elitist preservation, fitness reuse, and diversified candidate expansion. First, two groups of control experiments with and without elitist preservation are designed to verify the effectiveness of elitist preservation. The objective function uniformly adopts *f*_1_, and all other hyperparameters remain unchanged. The convergence curve and absorption spectra of candidates with elitist preservation are shown in Figure 3a and Figure 5a. The corresponding results without elitist preservation are presented in Figure 6a,b.

Comparisons show that the algorithm without elitist preservation stops converging at around the eighth generation. The minimum fitness stays at 0.12 and cannot be further optimized. Finer search in the local parameter space is not achieved in this case. In contrast, the algorithm with elitist preservation sustains intensive local search until the fitness converges to a minimum value of 0.08, yielding a superior absorber structure. The results reveal that elitist preservation can effectively enhance the local search capability and improve optimization precision.

The absorption spectra of candidate individuals without elitist preservation are plotted in Figure 6b. Compared with the scheme with elitist preservation shown in Figure 5a, the strategy without elitist preservation achieves acceptable absorption performance within the short-wave band of 0.36–4 μm. Nevertheless, a distinct absorption attenuation emerges in the long-wave band spanning 4–10.36 μm, where the absorptivity decreases to less than 90%, and absorption deviations and spectral fluctuations become obvious. The results in Table 2 further demonstrate that elitist preservation greatly improves the GA’s optimization performance and convergence stability, delivering superior structural parameters and enhanced broadband absorption.

FDTD simulation brings high computational cost. The fitness reuse strategy records fitness values of historical individuals. It avoids repeated simulations for individuals with identical parameters. Table 3 quantitatively evaluates the acceleration effect of this strategy in terms of the total number of simulations and total computation time. The theoretical total number (TTN) of simulations is 1550. The actual total number (ATN) of simulations drops obviously after fitness reuse is applied. The ATN accounts for approximately 55–61% of the theoretical value. The total computational time is nearly cut in half, and the optimization process is effectively accelerated. Results demonstrate that fitness reuse greatly reduces redundant calculations and cuts down computational resource consumption. The benefit becomes more significant for large-scale optimization tasks.

The best fitness individual may not optimally satisfy the engineering requirements in ultra-broadband MA design. This limitation arises from the intrinsic property of the objective function. The absorption spectra presented in Figure 5c and quantitative metrics in Table 1 provide support for this view. Some suboptimal fitness individual shows more advantages in multi-index trade-off. Case 1 and Case 2 under the hybrid objective function serve as a typical example. Case 1 has lower fitness, while Case 2 achieves superior performance in $\bar{A}$ and HAR. The HAR of Case 2 increases by 12 percentage points compared with Case 1. Furthermore, the discrepancies in spectral uniformity and deviation metrics between the two cases are negligible. Case 2 thus carries greater weight in practical engineering selection even though it does not have the best fitness value.

These results illustrate that diversified candidate expansion effectively extends the range of superior candidate solutions. It makes up for the limitations of the objective function. This offers more alternatives for engineering decision-making while enabling a comprehensive trade-off among bandwidth, absorptivity, and spectral uniformity.

### 3.3. Polarization, Angular, and Azimuthal Stability

In practical scenarios, incident EM waves are generally incoherent, unpolarized, and randomly oriented. Therefore, the MA is required to exhibit polarization insensitivity, wide-angle stability, and azimuthal independence. The effects of polarization state, incident angle, and azimuthal angle on the absorption of MA are systematically analyzed.

**Figure 7:**
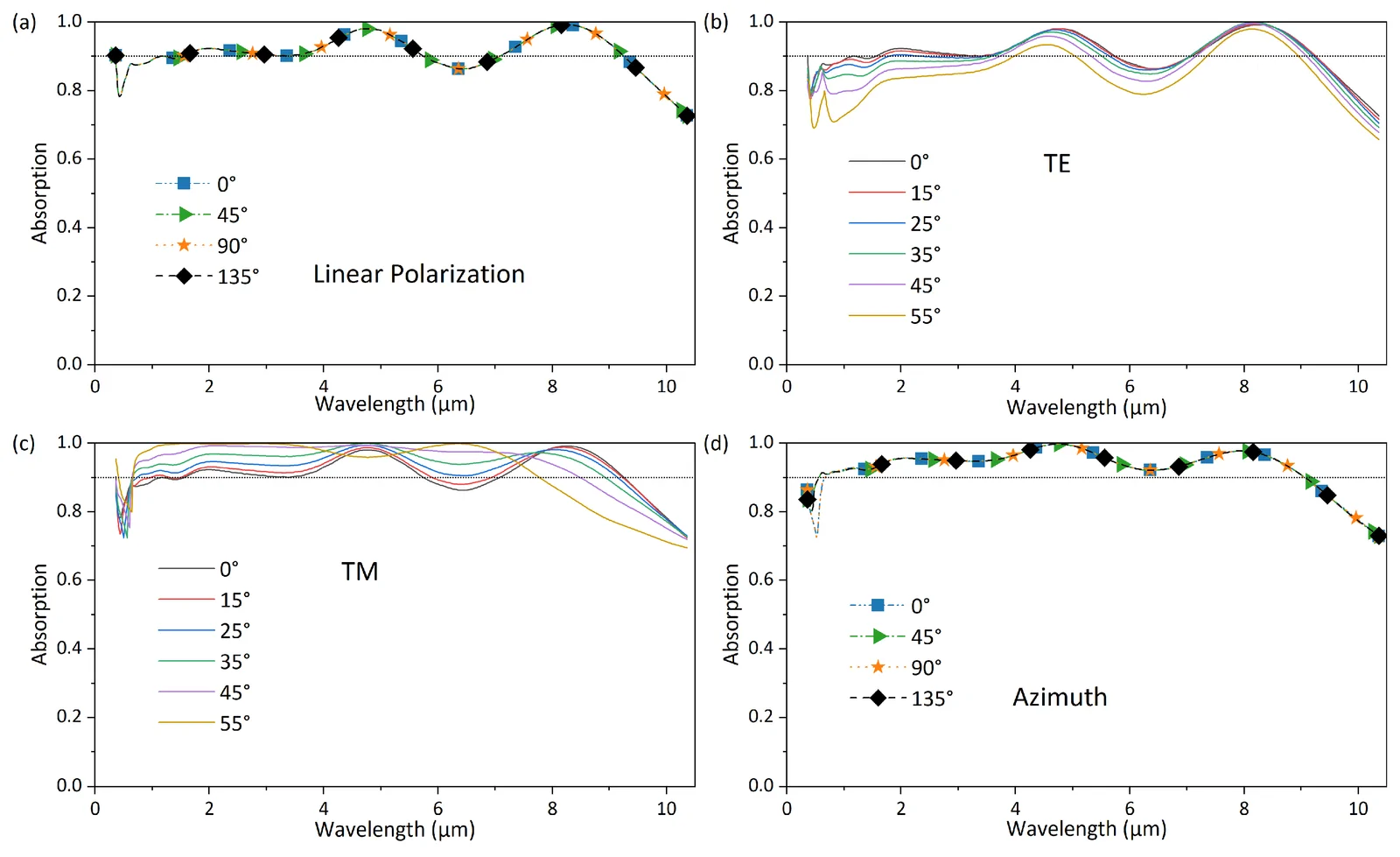
(**a**) Absorption spectra under normal incidence with different polarization angles. (**b**) Absorption spectra for TE polarization (*φ* = 0°) at different oblique incident angles. (**c**) Absorption spectra for TM polarization (*φ* = 0°) at different oblique incident angles. (**d**) Dependence of absorption spectra on azimuthal angle for TM polarization at 30° incident angle.

When the source is normal incidence with azimuth *φ* = 0°, linearly polarized excitations along *x*-direction (0°), 45°, *y*-direction (90°), and 135° are applied separately. Simulation results are presented in Figure 7a. The absorption curves under different polarization directions, o, nearly coincide with each other. The device exhibits ideal linear polarization insensitivity under normal incidence.

When the source is oblique incidence with the incident plane set as the *xz* plane and *φ* = 0°. Absorption responses under TE and TM polarizations are analyzed at different incident angles, *θ*. The electric field of TE polarization is perpendicular to the incident plane and points along the *y*-direction. The electric field of TM polarization is parallel to the incident plane and lies in the *xz* plane. Incident angles of 0°, 15°, 25°, 35°, 45°, and 55° are selected. A BFAST source is used in simulations to obtain broadband oblique incidence spectra.

Results are shown in Figure 7b,c. The overall absorption spectrum under TE polarization decreases gradually with increasing incident angle, and the absorption performance declines slightly. In contrast, the overall absorption spectrum under TM polarization rises gradually with increasing incident angle, and the absorption performance improves slightly. Neither polarization shows drastic absorption fluctuations over the full angle range. Stable high absorption is maintained in both cases. Table 4 shows that, under the polarization of TE and TM, the average absorptivity reaches 88.9% and 92.5% in the angle range of 0–55°, respectively. The unpolarized average absorptivity (*A*_unpol_ = (*A*_TE_ + *A*_TM_)/2) for incident angles of 0°, 15°, 25°, 35°, 45°, and 55° is 90.9%, 91.1%, 91.3%, 91.4%, 90.8%, and 88.6%, respectively. The overall unpolarized average absorptivity achieves 90.7%. These results demonstrate that the device maintains stable and high absorption for both TE and TM polarizations within a wide incident angle range of 0–55°.

The designed unit does not possess infinite-order rotational symmetry. Its resonant modes may be weakly modulated by the incident azimuthal angle, *φ*. Simulations are performed under TM polarization, with a fixed incident angle *θ* = 30°. Azimuthal angles (*φ*) of 0°, 45°, 90°, and 135° are adopted separately. Results are plotted in Figure 7d. The absorption spectrum shows no obvious change with varying azimuthal angles. Only weak differences appear in the short-wave region. Curves in the long-wave band almost completely overlap, indicating that the MA is insensitive to variations in the azimuth and exhibits good robustness to incident directions.

### 3.4. Absorption Mechanism

The physical mechanism underlying broadband absorption is analyzed using impedance matching theory. The normalized impedance of free space is 1, and ideal matching corresponds to Re(*Z_in_*) ≈ 1 and Im(*Z_in_*) ≈ 0. The input impedance, *Z_in_*, of the MA is calculated by Equation (9).

(9) $$Z_{in}=\frac{1+S_{11}}{1-S_{11}},$$

**Figure 8:**
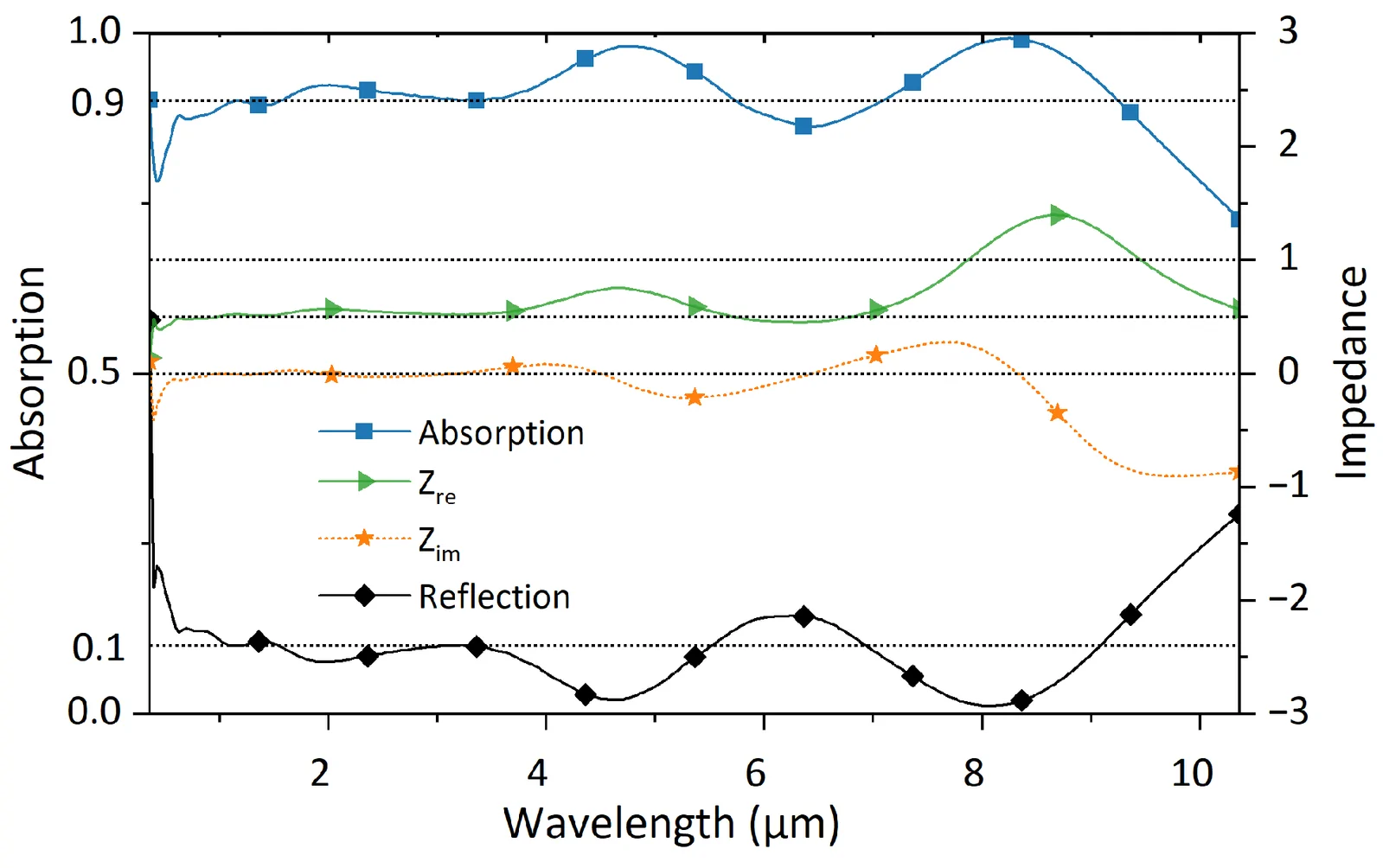
Broadband absorptivity, real and imaginary parts of equivalent impedance, and reflectivity. The reflectivity curve is calculated from Equation (12).

Taking case 2 of *f*_3_ as an example, the calculated results are presented in Figure 8. *Z*_re_ and *Z*_im_ represent Re(*Z_in_*) and Im(*Z_in_*), respectively. The results show that the *Z*_im_ remains close to zero over a broad spectral range, whereas *Z*_re_ is around 0.5. These results indicate that the broadband high absorption can still be achieved when the *Z*_re_ deviates from 1 only if the *Z*_im_ is close to 0. The metallic substrate thickness exceeds the skin depth, so the transmittance, *T*, is approximately zero. The absorptivity is thus determined solely by reflectivity, and the reflection coefficient, *r*, is calculated by Equation (10) [26,27].

(10) $$r=\frac{Z_{in}-Z_{0}}{Z_{in}+Z_{0}}$$

*Z*_0_ represents the impedance of free space. Reflectivity *R* is defined as $R={\left|r\right|}^{2}$ [27]. The input impedance is written as *Z*_in_ = *Z*_re_ + *j* · *Z*_im_. Then *R* can be expressed in the form of Equations (11) and (12).

(11) $$R={\left|\frac{Z_{re}+j\cdot Z_{im}-1}{Z_{re}+j\cdot Z_{im}+1}\right|}^{2}$$

(12) $$R=\frac{{\left(Z_{re}-1\right)}^{2}+Z_{im}^{2}}{{\left(Z_{re}+1\right)}^{2}+Z_{im}^{2}}$$

When *Z*_im_ = 0 and *Z*_re_ = 0.5, Equation (12) gives R ≈ 0.11, corresponding to relatively low reflection and high absorption. This example shows that high absorption may still be obtained when *Z*_re_ is not exactly equal to 1 if *Z*_im_ remains close to zero. Thus, the broadband absorption response should be understood in terms of the combined influence of Re(*Z_in_*) and Im(*Z_in_*). In the present structure, the relatively small Im(*Z_in_*) over a broad spectral range is an important characteristic associated with the low-reflection response. The black curve in Figure 8 shows the broadband reflectivity calculated by Equation (12). As expected, the overall reflectance maintains a low level, consistent with the simulated absorption spectrum. Impedance matching analysis reveals that the ultra-broadband absorption of the MA mainly originates from the joint contribution of Re(*Z_in_*) and Im(*Z_in_*) to the impedance matching between the MA and free space.

## 4. Discussion

### 4.1. Engineering Meaning of the Optimization Framework

The results show that the objective function does more than determine the numerical fitness value; it defines the design preference of the optimization process. Minimizing average reflectance favors a high overall absorption level but can allow spectral degradation at longer wavelengths, whereas minimizing absorptivity standard deviation favors spectral uniformity but does not necessarily maintain a high absorption level. The hybrid objective function therefore provides a practical compromise between absorption magnitude and spectral uniformity. This trade-off is important for practical applications, where a single peak absorption value is insufficient to describe the usefulness of the device. The multidimensional metrics used in this work further show that the lowest-fitness individual is not always the most attractive engineering candidate. Candidate diversification therefore complements the insufficient of single solution by preserving several high-quality solutions for subsequent engineering selection.

### 4.2. Roles and Limitations of the Three GA Strategies

The three strategies address different aspects of the inverse-design process. Elitist preservation reduces the risk that high-quality solutions are lost during stochastic genetic operations and allows the search to continue around promising regions. Fitness reuse reduces repeated FDTD calculations when an identical parameter combination is generated again. Candidate diversification retains multiple distinct solutions when the fitness cannot fully represent all engineering requirements. These strategies address search stability, computational redundancy, and engineering decision-making, respectively.

To examine whether the observed benefit of elitist preservation is robust to stochastic variation, five independent runs are used with the same hyperparameters. The best fitness of each generation is provided in Table 5. The mean and standard deviation of the final best fitness values over five independent runs with elitist preservation are 0.084 and 0.0049, respectively, whereas those without elitist preservation are 0.122 and 0.0299, respectively. These results indicate that the elitist preservation strategy improves the optimization performance of the genetic algorithm, resulting in a lower mean final best fitness. In addition, the smaller standard deviation demonstrates enhanced stability and consistency across independent runs. These results are in agreement with the conclusions drawn from the single-run optimization analysis.

Fitness reuse is particularly suitable for GA because selection, crossover, and mutation do not guarantee a completely new population in every generation. When an identical parameter vector is regenerated, the previously evaluated FDTD fitness can be reused. The fixed two-decimal representation of the geometric variables provides an identity criterion for the simulated geometry. For larger GA tasks with the same crossover and mutation probabilities, the potential saving can increase with population size and the number of generations. However, the benefit depends on the candidate-generation mechanism and should not be generalized to optimization algorithms with fundamentally different search mechanisms.

### 4.3. Comparison with Ref. [23]

Ref. [23] and the present study share the platform and the GA-assisted design. However, their methodological objectives are different. Ref. [23] focuses on extracting simplified physical relationships from parameter sweeps and electromagnetic-field analysis and incorporating these relationships as physical constraints into the GA. Its physical-model-constrained optimization can reduce or even remove the need for FDTD during the optimization stage. In contrast, the present study retains FDTD as the electromagnetic evaluator and develops an engineering-oriented GA inverse-design framework that jointly considers the optimization objective, search stability, repeated simulation cost, candidate diversity, and multidimensional engineering evaluation. The direct comparison is summarized in Table 6. The main contribution of this work is an engineering-oriented workflow that integrates multiple optimization and post-optimization strategies to generate and evaluate multiple candidate designs using comprehensive performance metrics.

### 4.4. Fabrication Feasibility

The feasibility of fabricating related nanoscale pyramidal structures has been demonstrated experimentally. Park et al. reported template stripping for refractory metals, oxides, and demonstrated fabricated tungsten nanopyramid arrays, providing a practical process basis for structures of the present type [28]. Wang et al. experimentally fabricated and characterized a pyramidal metamaterial absorber and reported favorable polarization and wide-angle optical behavior, providing relevant experimental evidence for related symmetric pyramidal absorber architectures [29]. These studies can be used as background evidence for fabrication and optical robustness of related structures.

## 5. Conclusions

This work establishes an engineering-oriented GA inverse-design framework for ultra-broadband metasurface absorbers by integrating a composite spectral objective with dynamic elitist preservation, fitness reuse, and diversified candidate screening. The optimized Zr/Fe_2_O_3_/Zr structure achieves broadband absorption over 0.36–10.36 μm, with an average absorptivity of 90.9%, a standard deviation of 0.054, and a coefficient of variation of 0.059. The framework also retains multiple high-quality candidate solutions, allowing engineering trade-offs to be considered beyond the single minimum-fitness solution. The present results demonstrate the numerical potential of the proposed design framework; however, experimental fabrication and characterization are still required. Overall, the results indicate that these strategies can be combined according to specific engineering requirements, while independent repeated runs can be used to assess stochastic robustness. Their applicability to other optimization algorithms should be evaluated according to the underlying candidate-generation mechanisms.

## Figures and Tables

**Table 1:** Multidimensional quantitative evaluation of candidate individuals optimized by *f*_1_, *f*_2_, and *f*_3_.

*f*/Case	$\bar{A}$	$\sigma_{A}$	CV	HAR	AD	MAD	RMSE	MRD	RD_max_
*f*_1_/1	0.921	0.071	0.077	0.830	9.389	0.110	0.133	0.123	0.268
*f*_1_/2	0.893	0.076	0.086	0.623	14.632	0.077	0.112	0.086	0.292
*f*_1_/3	0.878	0.105	0.119	0.699	22.823	0.151	0.186	0.168	0.373
*f*_1_/4	0.875	0.132	0.151	0.705	29.562	0.200	0.232	0.222	0.432
*f*_1_/5	0.872	0.090	0.103	0.493	21.205	0.083	0.128	0.093	0.353
*f*_1_/6	0.830	0.201	0.242	0.637	55.312	0.304	0.339	0.338	0.564
*f*_1_/7	0.830	0.080	0.096	0.084	35.693	0.078	0.111	0.086	0.352
*f*_1_/8	0.775	0.156	0.201	0.361	73.851	0.231	0.245	0.256	0.448
*f*_1_/9	0.766	0.174	0.227	0.307	76.623	0.221	0.260	0.245	0.585
*f*_1_/10	0.756	0.184	0.243	0.399	80.054	0.266	0.298	0.296	0.471
*f*_2_/1	0.862	0.053	0.061	0.204	23.213	0.058	0.069	0.065	0.172
*f*_2_/2	0.860	0.054	0.062	0.180	23.582	0.057	0.071	0.064	0.225
*f*_2_/3	0.862	0.055	0.064	0.218	23.600	0.060	0.072	0.067	0.204
*f*_2_/4	0.853	0.058	0.068	0.190	27.774	0.068	0.079	0.076	0.224
*f*_2_/5	0.853	0.060	0.070	0.190	28.419	0.070	0.080	0.078	0.213
*f*_2_/6	0.851	0.060	0.071	0.190	29.480	0.073	0.082	0.081	0.202
*f*_2_/7	0.845	0.062	0.073	0.164	31.284	0.075	0.088	0.083	0.223
*f*_2_/8	0.851	0.064	0.075	0.192	30.064	0.074	0.085	0.082	0.231
*f*_2_/9	0.831	0.064	0.077	0.170	38.297	0.092	0.101	0.102	0.255
*f*_2_/10	0.792	0.075	0.095	0.074	54.296	0.117	0.136	0.130	0.290
*f*_3_/1	0.906	0.046	0.051	0.527	7.483	0.032	0.043	0.035	0.146
*f*_3_/2	0.909	0.054	0.059	0.645	7.813	0.044	0.063	0.049	0.193
*f*_3_/3	0.904	0.056	0.062	0.593	10.228	0.050	0.063	0.056	0.182
*f*_3_/4	0.900	0.058	0.064	0.573	11.534	0.054	0.066	0.060	0.168
*f*_3_/5	0.895	0.060	0.067	0.467	13.013	0.049	0.063	0.054	0.164
*f*_3_/6	0.891	0.061	0.069	0.409	14.796	0.050	0.065	0.056	0.188
*f*_3_/7	0.894	0.079	0.088	0.571	15.436	0.072	0.103	0.080	0.307
*f*_3_/8	0.885	0.070	0.080	0.393	17.839	0.059	0.079	0.065	0.247
*f*_3_/9	0.898	0.090	0.100	0.659	16.837	0.098	0.135	0.109	0.322
*f*_3_/10	0.869	0.092	0.106	0.389	25.203	0.082	0.116	0.092	0.342

**Table 2:** Multidimensional quantitative evaluation of candidate individuals optimized by *f*_1_ without elitist preservation.

F/Case	$\bar{A}$	$\sigma_{A}$	CV	HAR	AD	MAD	RMSE	MRD	RD_max_
*f*_1_/1	0.877	0.101	0.115	0.531	27.593	0.117	0.133	0.130	0.276
*f*_1_/2	0.862	0.113	0.131	0.493	34.342	0.135	0.154	0.150	0.313
*f*_1_/3	0.857	0.119	0.139	0.491	36.880	0.145	0.164	0.161	0.342
*f*_1_/4	0.854	0.131	0.154	0.501	38.547	0.154	0.185	0.171	0.443
*f*_1_/5	0.853	0.120	0.141	0.489	37.938	0.148	0.169	0.165	0.359
*f*_1_/6	0.851	0.121	0.142	0.485	38.605	0.150	0.170	0.166	0.286
*f*_1_/7	0.850	0.123	0.145	0.471	39.481	0.149	0.171	0.166	0.296
*f*_1_/8	0.849	0.124	0.146	0.489	38.980	0.152	0.177	0.169	0.304
*f*_1_/9	0.838	0.134	0.159	0.481	44.144	0.170	0.196	0.189	0.331
*f*_1_/10	0.837	0.135	0.162	0.413	45.872	0.156	0.186	0.173	0.327

**Table 3:** Optimization cost of the improved genetic algorithm.

Case	*f*	Elitism	TTN	ATN	ATN/TTN	Time/min
1	*f*_1_	Yes	1550	891	0.57	183
2	*f*_1_	No	1550	940	0.61	162
3	*f*_2_	Yes	1550	845	0.55	171
4	*f*_3_	Yes	1550	870	0.56	176

**Table 4:** Average absorptivity at various incident angles under TE and TM polarizations.

*Θ*	*A*_TE_ (%)	*A*_TM_ (%)	*A*_unpol_ (%)
0	90.9	90.9	90.9
15	90.7	91.5	91.1
25	90.3	92.4	91.3
35	89.3	93.5	91.4
45	87.7	94.0	90.8
55	84.7	92.6	88.6
Avg (%)	88.9	92.5	90.7

**Table 5:** Multiple-run validation of the effectiveness of elitist preservation.

Gen	NE 1	NE 2	NE 3	NE 4	NE 5	E 1	E 2	E 3	E 4	E 5
0	0.23	0.19	0.28	0.2	0.26	0.19	0.27	0.27	0.18	0.22
1	0.17	0.18	0.23	0.2	0.26	0.17	0.17	0.24	0.18	0.22
2	0.17	0.18	0.22	0.17	0.25	0.17	0.17	0.19	0.18	0.22
3	0.17	0.15	0.18	0.16	0.21	0.14	0.16	0.17	0.15	0.18
4	0.16	0.15	0.14	0.16	0.21	0.13	0.13	0.16	0.15	0.18
5	0.16	0.15	0.14	0.16	0.19	0.12	0.11	0.13	0.15	0.18
6	0.16	0.14	0.14	0.13	0.2	0.1	0.1	0.13	0.15	0.11
7	0.15	0.13	0.14	0.13	0.19	0.1	0.1	0.12	0.11	0.11
8	0.13	0.13	0.13	0.13	0.19	0.1	0.1	0.09	0.1	0.11
9	0.13	0.13	0.12	0.13	0.19	0.1	0.09	0.09	0.1	0.1
10	0.13	0.13	0.11	0.13	0.18	0.1	0.09	0.09	0.1	0.08
11	0.13	0.12	0.11	0.13	0.18	0.1	0.09	0.09	0.09	0.08
12	0.13	0.12	0.11	0.13	0.18	0.1	0.09	0.09	0.09	0.08
13	0.13	0.12	0.11	0.13	0.18	0.09	0.08	0.09	0.09	0.08
14	0.13	0.11	0.11	0.13	0.18	0.09	0.08	0.09	0.09	0.08
15	0.13	0.11	0.1	0.12	0.18	0.09	0.08	0.09	0.09	0.08
16	0.13	0.11	0.1	0.12	0.18	0.09	0.08	0.09	0.09	0.08
17	0.13	0.11	0.1	0.12	0.18	0.09	0.08	0.09	0.09	0.08
18	0.12	0.11	0.1	0.12	0.18	0.09	0.08	0.09	0.09	0.08
19	0.12	0.11	0.1	0.12	0.18	0.09	0.08	0.09	0.09	0.08
20	0.12	0.11	0.1	0.12	0.18	0.09	0.08	0.09	0.09	0.08
21	0.12	0.1	0.1	0.12	0.18	0.09	0.08	0.09	0.09	0.08
22	0.12	0.1	0.1	0.12	0.18	0.09	0.08	0.09	0.09	0.08
23	0.12	0.1	0.1	0.12	0.18	0.09	0.08	0.09	0.09	0.08
24	0.12	0.1	0.1	0.12	0.18	0.09	0.08	0.09	0.09	0.08
25	0.11	0.1	0.1	0.12	0.18	0.09	0.08	0.09	0.09	0.08
26	0.11	0.1	0.1	0.12	0.18	0.09	0.08	0.09	0.09	0.08
27	0.11	0.1	0.1	0.12	0.18	0.08	0.08	0.09	0.09	0.08
28	0.11	0.1	0.1	0.12	0.18	0.08	0.08	0.09	0.09	0.08
29	0.11	0.1	0.1	0.12	0.18	0.08	0.08	0.09	0.09	0.08
30	0.11	0.1	0.1	0.12	0.18	0.08	0.08	0.09	0.09	0.08

**Table 6:** Direct comparison between Ref. [23] and the present study.

Aspect	Ref. [23]	Present Study
Architecture and materials	Pyramid, Zr/Fe_2_O_3_/Zr	Pyramid, Zr/Fe_2_O_3_/Zr
Objective/fitness	Physical-model-constrained objective based on simplified absorption relationships and penalty terms	Composite objective based on full-spectrum average reflectance and absorptivity standard deviation
Optimization variables	Aspect ratio, *A*; lateral duty cycle, *D*; top-shape parameter, *S*; and edge slope, *K*	*W_b_*, *W_t_*, *H*, and *T*_2_
GA implementation	GA with physical-model constraints; physical model can operate without FDTD	FDTD-driven GA with dynamic elitist preservation, fitness reuse, and diversified candidate screening
Spectral performance	Average absorption about 90% over 0.35–6 μm	Average absorptivity of 90.9% over 0.36–10.36 μm
Computational efficiency	Main advance is reducing FDTD dependence through physical-model constraints	Main advance is reducing redundant FDTD calculations and improving search stability and candidate selection
Polarization/angular analysis	Polarization stability and temperature dependence were numerically examined	TE/TM absorption evaluated from 0° to 55°, and azimuthal dependence also examined
Physical interpretation	Physical constraints derived from parameter–response relationships and field analysis guide the GA	Broadband response interpreted using impedance matching together with objective-function and candidate-level engineering analysis

## Data Availability

The original contributions presented in this study are included in the article. Further inquiries can be directed to the corresponding author.
